# Contribution of Non-*Saccharomyces* Yeasts to Wine Freshness. A Review

**DOI:** 10.3390/biom10010034

**Published:** 2019-12-25

**Authors:** Antonio Morata, Carlos Escott, María Antonia Bañuelos, Iris Loira, Juan Manuel del Fresno, Carmen González, José Antonio Suárez-Lepe

**Affiliations:** 1 enotecUPM, Department of Chemistry and Food Technology, Universidad Politécnica de Madrid, 28040 Madrid, Spain; carlos.escott@gmail.com (C.E.); iris.loira@upm.es (I.L.); juanmanuel.delfresno@upm.es (J.M.d.F.); carmen.gchamorro@upm.es (C.G.); joseantonio.suarez.lepe@upm.es (J.A.S.-L.); 2 enotecUPM, Department of Biotecnology, Universidad Politécnica de Madrid, 28040 Madrid, Spain; mantonia.banuelos@upm.es

**Keywords:** non-*Saccharomyces*, wine, yeast metabolites, biopolymers, sensory quality, freshness

## Abstract

Freshness, although it is a concept difficult to define in wines, can be understood as a combination of different circumstances. Organolepticwise, bluish red, floral and fruity, more acidic and full-bodied wines, are perceived as younger and fresher by consumers. In traditional winemaking processes, these attributes are hard to boost if no other technology or biotechnology is involved. In this regard, the right selection of yeast strains plays an important role in meeting these parameters and obtaining wines with fresher profiles. Another approach in getting fresh wines is through the use of novel non-thermal technologies during winemaking. Herein, the contributions of non-*Saccharomyces* yeasts and emerging technologies to these parameters are reviewed and discussed.

## 1. Introduction

Wine freshness is a complex concept comprising mouth, smell and visual perceptions [1]. In this regard, the freshness of the wine could be defined by the combination of a fruity aroma reminiscent of the grape variety, moderate ethanol content and high acidity. Therefore, it is not only a question of achieving a refreshing flavour in the wine, but also of preserving the typicity of the grape variety. The freshness of wine can be reached from different microbiological perspectives, for instance, by using different non-*Saccharomyces* yeasts that produce organic acids [2]. It can also be reached through the ability of microorganisms, yeasts and bacteria, that synthesise and subsequently free enzymes capable of releasing volatile thiols (e.g., 3-mercaptohexan-1-ol and 3-mercaptohexyl acetate) or terpenes (e.g., geraniol, linalool) [3], thus contributing to the fresh aroma of the wine. Similarly, the freshness of the wine could also be correlated to some extent with increased production of acetate esters [4].

Fruity and floral aroma are responsible for wine aromatic freshness and they are strongly connected with the production of acetate esters from higher alcohols or short chain fatty acids ethanolic esters during fermentation [5]. Selected *Saccharomyces* strains can help to improve the concentrations of these compounds, but especially several non-*Saccharomyces* species are able to increase them significantly during fermentation. Among them, *Torulaspora delbrueckii* (Td) [6], *Wickerhamomyces anomalus* (Wa) [7], *Metschnikowia pulcherrima* (Mp) [8], *Hanseniaspora vineae* (Hv) and *Hanseniaspora*/*Kloeckera* spp. [9], *Lachancea thermotolerans* [2,10], or *Candida stellata* (Cs) [11] have proven their effectiveness. Some of them also have the ability to release varietal aroma from precursors such as glycosylated terpenes or bonded thiols by means of *β*-glucosidase [12] or *C-S*-lyase activities [13]. Many of these yeast species are commercialised as dry or frozen products, while others will be available in the near future. Moreover, the number of available selected strains of these species is progressively growing in the market. 

Concerning the sense of taste, freshness is strongly connected with acidity. Acid wines are perceived as more refreshing than low acidity-high pH wines which frequently show heavy and winey profiles. Acidity can be improved by a biological way during fermentation. Some yeasts are able to produce malic acid and lactic acid affecting wine’s pH, but also some other acids in lower concentrations such as succinic acid, pyruvic acid, etc., without significant repercussions to the pH values. The production of malic acid is quite typical in many strains of *Saccharomyces cerevisiae* (Sc), however, the production is low, usually below 1 g/L [14]. Malic acid is unstable because it can be metabolised by lactic acid bacteria and its technical utility is quite low. Conversely, lactic acid is an interesting option because some non-*Saccharomyces* yeasts as *Lachancea thermotolerans* (Lt) can produce it in high concentration [2], even in oenological conditions at a variable range of pH [15]. Moreover, this acid is stable along the winemaking processes and its sensory perception is good [1] and, unlike general belief, it can be described as citric fruit acidity without dairy notes [15].

Freshness can also be related to colour. Red-brown hues make consumers perceive wines as oxidised, therefore colour hue and intensity influence the sensory profile. Yeast can affect colour in several aspects [16]: (i) production of organic acids with repercussion on pH increases the colour of anthocyanins by hyperchromic effect, but it also affects colour stability [2], (ii) the release of yeast metabolites or the expression of hydroxycinnamate decarboxylase activities promote the formation of stable pigments like vitisin or vinylphenolic adducts, respectively [17,18], (iii) the enhancement of polymeric pigments formation [19,20], (iv) the removal of anthocyanins by cell walls adsorption during fermentation [21,22,23], and (v) the production of reductive compounds as glutathione (GSH) with protective effects on wine anthocyanins [2]. The use of some non-*Saccharomyces* species has proven effective in colour protection through pH modification or the formation of stable pyranoanthocyanins or polymeric pigments.

Wine structurewise, there is a release of yeast polysaccharides during fermentation and also during ageing on lees (AOL). AOL is an interesting technique that involves contact between the lees and the wine after fermentation. In addition, AOL protects the fruity aroma due to its reductive properties [24]. The use of non-*Saccharomyces* is a current biotechnology to increase the polysaccharide content and to speed up the AOL process [25,26]. Moreover, emerging technologies such as ultrasounds [27,28,29] or ultra-high pressure homogenization [30] facilitate the release of yeast cell wall polysaccharides during AOL. Nonetheless, the use of AOL counteracts freshness by modulating the acidity.

**Figure 1:**
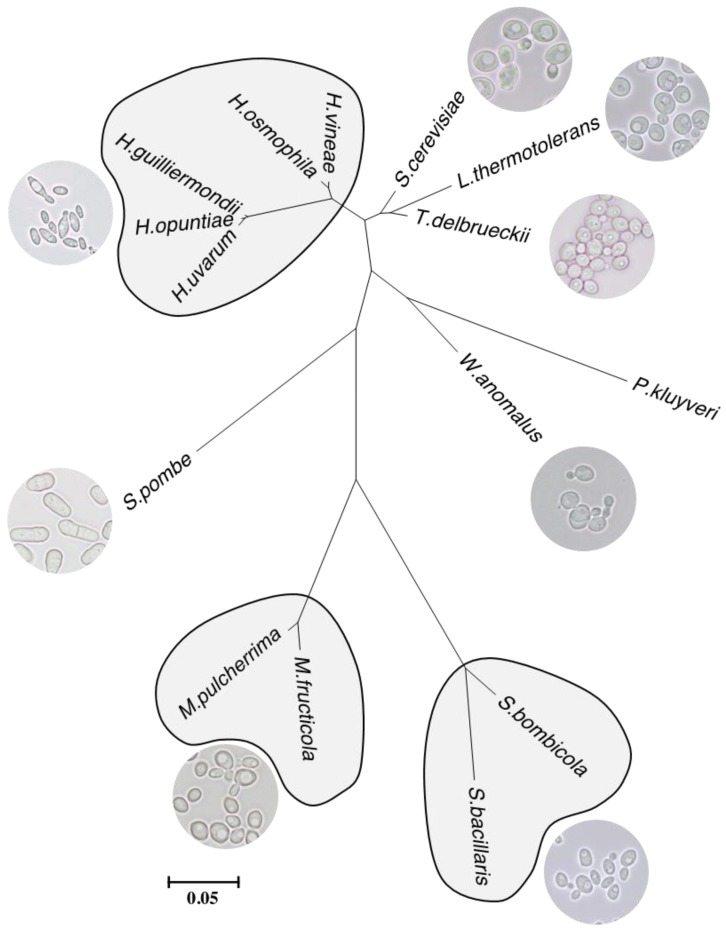
Phylogenetic relationships among wine yeast species based on analysis of D1/D2 LSU rRNA gene sequences. The evolutionary history was inferred by using the Maximum Likelihood method based on the Tamura-Nei model in MEGA7. GenBank accession numbers follow strain numbers: *Saccharomyces cerevisiae* NRRL Y-12632/AY048154; *Metschnikowia pulcherrima* NRRL Y-7111/U45736 *Metschnikowia fructicola* B-4(1)/EU441890; *Lachancea thermotolerans* CBS 2803/KY108273; *Torulaspora delbrueckii* NRRL Y-866/U72156; *Wickerhamomyces anomalus* NRRL Y-366/U74592; *Pichia kluyveri* NRRL Y-11519/U75727; *Hanseniaspora uvarum* NRRL Y-1614/U84229; *Hanseniaspora opuntiae* CBS 8733/AJ512453; *Hanseniaspora vineae* NRRL Y-17529/U84224; *Hanseniaspora osmophila* NRRL Y-1613/U84228; *Hanseniaspora guilliermondii* NRRL Y-1625/U84230; *Schizosaccharomyces pombe* NRRL Y-12796/AY048171; *Starmerella bombicola* 16-D-2/KF935227; *Starmerella bacillaris* CBS 1713/KY109779.

The classification of some non-*Saccharomyces* used in wine biotechnology can be done by molecular techniques according to their phylogenetic relations. The classification of yeast species and genera was formerly assigned from phenotypical analysis in function of morphology of vegetative cells and sexual states, and physiological responses in growth media and fermentation essays; at present, this classification is given by gene sequencing [31] or genotype, and this is the reason why yeasts species with similar phenotype are grouped in different clusters/branches of phylogenetic trees (Figure 1).

One of the main drawbacks in the oenological use of non-*Saccharomyces* is the difficulty in reaching suitable implantations in musts with native microflora. Thus, their metabolome expression is limited and so is the release of metabolites and the production of enzymatic activities with sensory repercussion. Emerging non-thermal technologies open the door to eliminate the wild microbiome from grapes allowing the suitable implantation of these non-*Saccharomyces* yeasts [32]. Many of these techniques such as high hydrostatic pressures, ultra-high pressure homogenization, β-irradiation, pulsed electric fields, etc., also have concomitant advantages as the enhancement of the extraction of volatile compounds and polyphenols from grapes and the inactivation of oxidative enzymes allowing the reduction in sulphites content.

## 2. Influence of Non-*Saccharomyces* Yeasts on Wine Aroma

**Figure 2:**
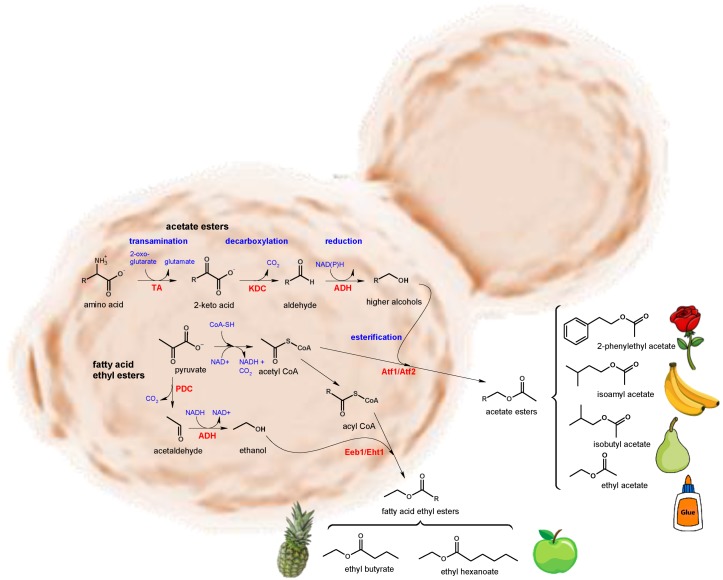
Metabolic pathways involved in the formation of floral and fruity esters in yeasts. Production of acetate esters by Ehrlich catabolism of amino acids and production of fatty acids ethyl esters (*TA:* transaminase, *KDC:* 2-keto acid decarboxylase, *ADH:* alcohol dehydrogenase, *Atf:* acyltransferase). Formation of fatty acid ethyl esters by acylation with acyl-CoA (PDC: pyruvate decarboxylase, ADH: alcohol dehydrogenase, Eeb1/Eht1: ethyl ester biosynthesis/ethanol hexanoyl transferase).

The formation of fruity or floral aroma during fermentation is an important contribution of non-*Saccharomyces* yeasts to wine freshness. Many esters with sensory impact are formed as a consequence of the amino acids catabolism by the Ehrlich transamination pathway (Figure 2). The higher alcohols synthesised in this process can be esterified with acetic acid to produce low threshold aromatic esters (e.g., 2-phenylethyl acetate, isoamyl acetate, isobutyl acetate), most of them with floral or fruity descriptors (e.g., rose petals, banana, pear). Moreover, several acids and short chain fatty acids (e.g., lactic, acetic, butyric) are also formed during the yeast metabolism. Some of them produce fruity or sweet profiles (e.g., strawberry, toffee), enhancing freshness and reducing the neutral and winey profile of the conventional *S. cerevisiae strains*. 

The production of aromatic compounds by yeasts has been previously reviewed in several key studies that highlight the role of the production of acetate esters from higher alcohols, fatty acid ethyl esters (Figure 2) and the enzymatic activities that release terpenes and thiolic compounds from cysteine-derivatives [5,33,34].

**Figure 3:**
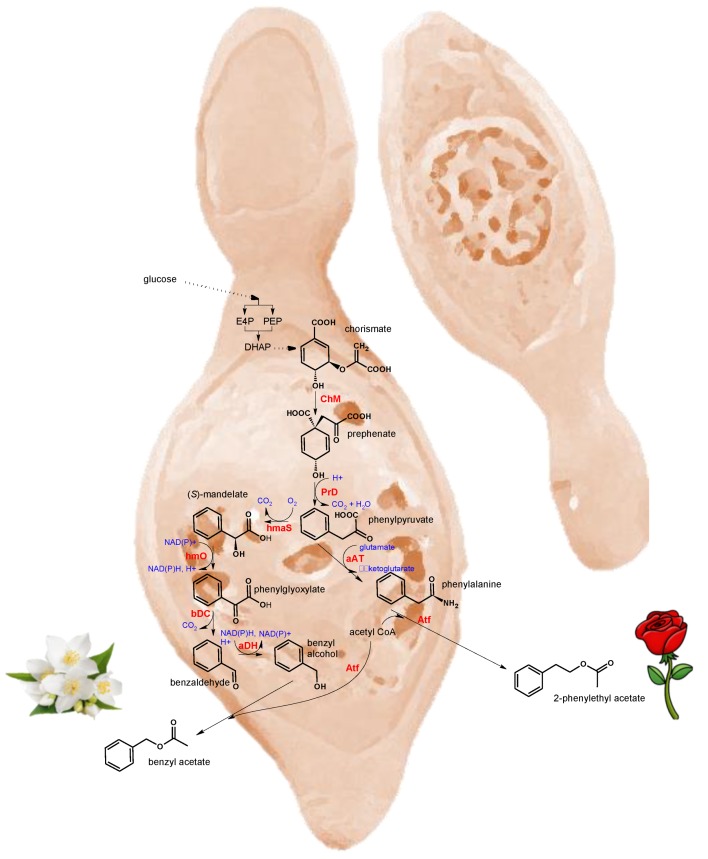
Metabolic pathways involved in the *de novo* synthesis from sugars of floral esters by *H. vineae* following the chorismate-prephenate-(*S*)-mandelate/phenylalanine pathway (*ChM:* chorismate mutase, PrD: prephenate dehydratase, *hmaS:* hydroxymandelate synthase, *hmO:* hydroxymandelate oxidase, *bDC:* benzoylformate decarboxylase, *aDH:* benzyl alcohol dehydrogenase; aAT: aromatic aminotransferase; Atf: acyltransferase). Adapted from [36,38,39].

Regarding the *H. vineae* species, the main esters with sensory repercussion from its exometabolome are benzyl acetate, 2-phenylethyl acetate, ethyl lactate and 3-methylbutyl acetate [35]. Among them, the first two esters are released in higher concentrations (2–7×) than *S. cerevisiae*; by comparison, ethyl lactate is usually produced below the sensory threshold. Formation *de novo* of benzyl acetate and 2-phenylethyl acetate from sugars by the chorismate-prephenate pathway has been reported ([36], Figure 3). It has been observed that nitrogen nutrition affected the formation of benzyl and 2-phenylethyl acetates. High doses of diammonium phosphate (100–250 mg/L) inhibited the production of these phenylpropanoid compounds and negatively affected the global balance of aromatic esters [37]. Benzyl alcohol, precursor of benzyl acetate, can be obtained *de novo* without using grape precursors by 11 different *H. vineae* strains at concentrations 20–200 fold higher than with *S. cerevisiae* strains [36].

Currently, *Torulaspora delbrueckii* is a non-*Saccharomyces* yeast with great oenological potential due to its good fermentation performance with low levels of ethanol, higher alcohols, volatile fatty acids and volatile acidity, and its notable contribution to the aromatic profile of the wine [6,40]. Some authors have found a synergistic effect between *T. delbrueckii* and *S. cerevisiae* when fermenting in co-inoculation with a high proportion of the non-*Saccharomyces* yeast, leading to enhanced aromatic properties in the wines [40,41]. In addition, certain volatile compounds contributed by *T. delbrueckii* have been positively related to the fruity character of the wine. For instance, linalyl acetate, with citrus aroma descriptor, far exceeded its odour threshold (0.05 mg/L) in white Muscat fermentations by *T. delbrueckii* [40]. Similarly, sequential fermentation between *T. delbrueckii* and *S. cerevisiae* resulted in higher concentrations of 3-mercaptohexan-1-ol (grape fruit and passion fruit descriptors [42]) and its acetate (boxwood and passion fruit descriptors [43]) [41]. 3-Ethoxy-1-propanol is another interesting volatile compound that is produced specifically by *T. delbrueckii* and can contribute to the fruity character of wine with a descriptor of blackcurrant aroma [44,45]. However, in most cases it is not possible to state with complete certainty that a non-*Saccharomyces* yeast is responsible for specific changes in the volatile profile (always in the same way), as other important factors may be involved such as the strain variability factor or the ratio of dominance against other fermentative yeasts, especially in comparison with *Saccharomyces cerevisiae* [6]. Therefore, it is of great interest to know those species or strains that tend to preserve the fresh-fruit aromatic intensity coming from the grape variety, as well as the working conditions under which these effects are enhanced. 

*Pichia kluivery* is another yeast species capable of releasing varietal aromas. Among these aroma precursors are thiol-type varietal aromas: 4-methyl-4-mercaptopentan-2-one (4M4MP-boxwood, broom), 3-mercaptohexanol (3MH-grapefruit), 3-mercaptohexyl acetate (3MHA-passion fruit) [42]. *P. kluivery* produces thiol aromas in a concentration similar to *S. cerevisiae* strains in single fermentations, but it is able to enhance the production of such compounds in co-inoculations with *S. cerevisiae* strains [46]. The production of 3MH increases from 625 ng/L to 3000 ng/L while the production of 3MHA increases from 500 ng/L to 1700 ng/L from single to co-inoculation. respectively.

*Metschnikowia pulcherrima*, like the aforementioned non-*Saccharomyces* yeast species, are also able to influence the aroma profile of wines and, therefore, to have an impact in the sensory quality of wines [47]. *M. pulcherrima* contributes to the volatile fraction with the production of aroma compounds due to its *β*-glucosidase activity [48]. In addition, some *M. pulcherrima* strains were also reported to have *β*-xylosidase activity [48,49] which increases the enzymatic activity during fermentation. In this regard, *M. pulcherrima* seems prone to releasing more monoterpenols and 2-phenyl ethanol than other yeast species such as *H. guilliermondii* [48]. Linalool, geraniol and nerol are among these monoterpenol compounds.

The following table summarises some of these metabolites mainly contributed by non-*Saccharomyces* yeasts (Table 1).

## 3. Acidity and pH Control

The effect of *L. thermotolerans* in wine freshness is especially significant because of the influence on pH by the production of lactic acid; some strains are capable of producing more than 16 g/L [52]. Recent reviews have highlighted the oenological relevance of this species [2,10]. The phenotypic and genotypic variability of strains from many origins worldwide have been studied [55,56]. The formation of lactic acid is derived from pyruvate in the glycolytic metabolism of sugars. The enzyme lactate dehydrogenase is involved in the reduction of pyruvate to lactic acid and it is a typical feature found in many strains of *L. thermotolerans* [15]. This acidification happens at the beginning of the alcoholic fermentation, 3–5 days of fermentation [2], with a significant effect when the population exceeds 6-log CFU/mL. Effective pH reductions have been reported in mixed fermentations with inoculation ratio 7-log:3-log (Lt:Sc) [57]. The early acidification helps *L. thermotolerans* to be competitive even when high wild populations are present in the must during fermentation. Nitrogen contents also affect the production of lactic acid; YAN values closed to 200 mg/L improve lactic acid production [2]. Strong effects in pH have been observed in Tempranillo red wines with initial pH ca. 4; lactic acid levels exceeded 6 g/L when specific *L. thermotolerans* strain was used and the pH value decreased around 0.5 units [15]. *L. thermotolerans* has been described as low volatile acidity producer, ranging most of the strains 0.3-0.5 mg/L [15,57,58], and also as an interesting bio-tool to control the acetic acid production [59]. Since lactic acid production can come from sugar depletion, there could be a slight reduction of alcoholic degree (0.3–0.5% vol.) [15]. Current literature describes fermentative performances of around 8–10% vol. [56,57], however, this can be improved with appropriate strain selection. Single pure fermentations with *L. thermotolerans*, isolated and selected in ongoing research works, might be possible since these strains are able to yield 12–13% vol. ethanol.

Lactic acid can be described as citric fruit acidity without providing the wines with dairy notes. The perceived effect is the enhancement of the freshness in mouth. Dairy notes resembling compounds, such as diacetyl or acetoin, are produced at similar concentration when used in sequential inoculations than in *S. cerevisiae* pure fermentations [15]. 

Even when non-*Saccharomyces* are described as low sulphite-resistant, many *L. thermotolerans* strains have the capacity to grow and ferment in the presence of sulphites. Some strains can ferment in the presence of conventional concentrations of 50–60 mg/L of total SO_2_ with a slight reduction in the final alcoholic degree. It is also possible to ferment at slower rate under 18 °C with 70 mg/L of total SO_2_ and 15 mg/L of free SO_2_ which resembles winemaking conditions.

## 4. Non-*Saccharomyces*-Mediated Formation of Stable Pyranoanthocyanin and Polymeric Pigments

Different yeast genera may contribute to the formation of stable pigments either during the fermentation or during the ageing period. In this regard, anthocyanins extracted from the grape’s skins are transformed through condensation reactions into less sensitive pigments against pH variations in wines, addition of SO_2_ to improve the wine’s stability and temperature fluctuations during the product’s shelf life. Among the species known for having this activity are *S. pombe* due to an increase of vitisin A formation [60]; *T. delbrueckii* and *M. pulcherrima* that favour the production of oligomeric fractions in sequential fermentations [19]; *L. thermotolerans* that is able to increase the amount of lactic acid during must fermentation [2], which pH reduction improves the colour intensity of wines and its stability by increasing the molecular SO_2_. It also adsorbs less pigments on the cell wall which produces higher concentration of final total pigments in sequential fermentations [20].

**Figure 4:**
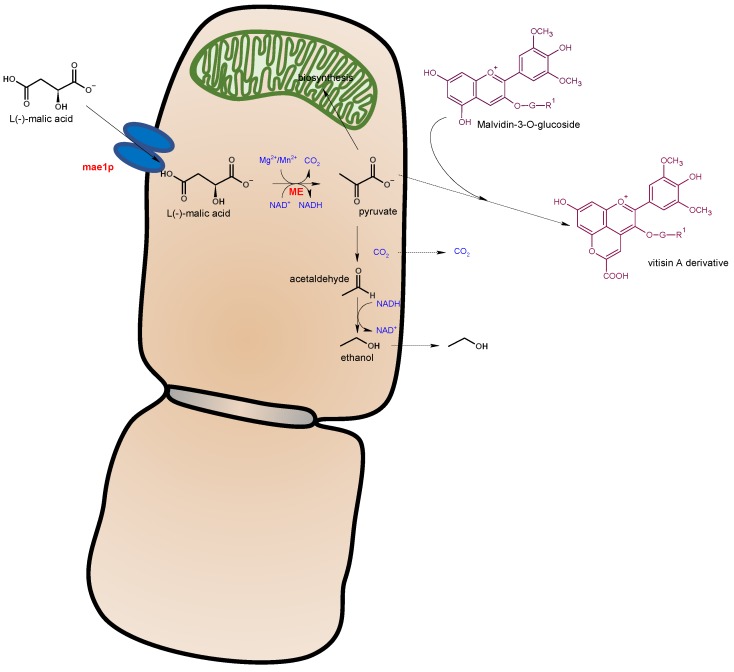
Maloalcoholic fermentation by *Schizosaccharomyces pombe* and its influence in the subsequent formation of stable vitisin A-type pigments by chemical condensation with grape anthocyanins (adapted from [17,61]).

Maloalcoholic fermentation (MAF) produces the metabolisation of malic acid into ethanol. The malic enzyme (ME) of *S. pombe* has 15-folds higher affinity by malic acid than the ME of *S. cerevisiae* [61]. This higher efficiency is due to the presence of the specific malate permease transporter in *S. pombe* (mae1p) facilitating the entrance of malate into the cytosolic media, and also because the ME is located in the cytosol conversely to *S. cerevisiae* in which this pathway is produced in the mitochondrion [61]. MAF of *S. pombe* increases the release of extracellular pyruvate facilitating the formation of stable vitisin A-type derivatives [62] by chemical condensation with grape anthocyanins [17] (Figure 4). The higher the extracellular release of pyruvate, the higher the formation of stable pyranoanthocyanins. Pyranoanthocyanins are more stable pigments under oenological conditions not only because of their lower sensibility to SO_2_ bleaching, but also to their lesser hypochromic pH effect [63].

**Figure 5:**
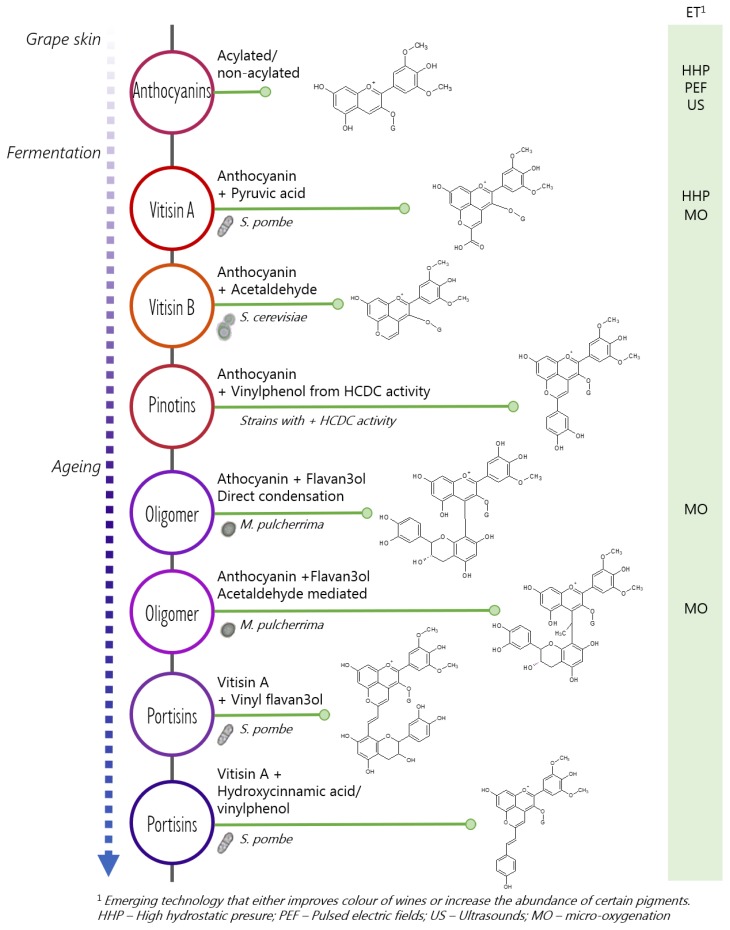
Example of red wine pigments and their occurrence during the different winemaking stages. From top to bottom the pigments shown in the figure are: malvidin-3-glucoside, malvidin-3-glucoside pyruvic acid (vitisin A), malvidin-3-glucoside-4-vinyl (vitisin B), malvidin-3-glucoside 4-vinylcatechol (pinotin A), dimer malvidin-3-glucoside-catechin, dimer malvidin-3-glucoside-ethyl-catechin, malvidin-3-glucoside-pyruvic acid vinyl catechin (portisin type A) and malvidin-3-glucoside pyruvic acid vinyl phenol (portisin type B). Yeasts species contributing to the formation of particular pigments are also indicated.

Besides pyruvic acid, acetaldehyde is another fermentative metabolite playing an important role in the formation of stable pigments in red wines. Such is the case of all the pyranoanthocynin-type pigments [18,64], and the oligomers condensed through ethyl bridges [65] (Figure 5). The formation of ethyl linked oligomers happens preferentially with (-)-epicatechin rather than with (+)-catechin in acidic conditions [66]. This mechanism is explained from the slower rate in which the condensation with the latter flavanol takes place. Nonetheless, the stability of oligomeric pigments varies with the absence/presence of acetaldehyde linkages and the configuration of the pigment. The configuration of anthocyanin-flavanol ethyl linked dimers is more stable than the configuration of flavanol-flavanol ethyl linked dimers [67], but both are apparently less stable than the dimers formed from direct condensation of anthocyanin-flavanol moieties [68] which takes place slower over time in aged wines.

Pinotins are pigments produced from the reaction of anthocyanins or pyranoanthocyanins with hydroxycinnamic acids or 4-vinyl phenols [69]; in this last case, the yeast strains with positive hydroxycinnamate decarboxylase activity can produce the intermediate compound that will condense via nucleophilic attack at position C4 of the anthocyanin structure [70]. Other pigments, mainly formed during the ageing period, comprise the previous formation of vitisins for further condensation with vinyl-flavan-3-ols and/or hydroxycinnamic acids or 4-vinyl phenols [71]; these reactions would produce the so called portisin-type pigments with bluish tonality due to an absorption at 570 nm [72] of the electromagnetic spectrum.

In the same way that yeast strains promote the formation of pigments, emerging preservation technologies for grape berries/must processing and winemaking could improve the colour in wines (Figure 5). Such is the case of the high hydrostatic pressure (HHP) that increases total anthocyanins extraction and the formation of pyranoanthocyanins [73], especially the amount of vitisin A; the use of pulsed electric fields (PEF) or the ultrasounds (US) that have shown to produce an increase in anthocyanin extraction from cell wall structures after suffering membrane disruption [74,75]; the micro-oxygenation (MO) could also have an impact in the colour of red wines through the formation of vitisins or direct condensation of anthocyanin and tannins where oxygen and reactive species may play an important role [76].

Other information related to the nature and bioavailability of anthocyanins, their properties and industrial applications as well as the formation of pyranoanthocyanins and polymeric pigments has been recently published in reviews [77,78,79,80]. 

## 5. Commercially Available Non-*Saccharomyces* Yeasts

The use of non-*Saccharomyces* yeasts has grown progressively from the initial applications of *T. delbrueckii* to enhance aromatic profile with two widely spread strains Biodiva^TM^ Td291 (Lallemand, Blagnac Cedex, France) and Prelude^TM^ (CHR Hansen, Hoersholm, Denmark), and also from the use of *S. pombe* reticulated in alginate beads for demalication (Proenol), *L. thermotolerans* (formerly *Kluyveromyces thermotolerans*) and *P. kluyveri* (CHR Hansen). Non-*Saccharomyces* yeasts are frequently offered mixed with *S. cerevisiae* to ensure the full depletion of the sugars and achieve wine dryness. Main commercial non-*Saccharomyces* yeast species were described by Morata and Suarez [81], and are shown in an updated list in Table 2.

Ternary inoculations have been proposed at industrial level by Chr. Hansen in the commercial blend of three stains, *S. cerevisiae* (60%), *T. delbrueckii* (20%) and *L. thermotolerans* (60%:20%:20%) known as Viniflora^®^ Melody™. This approach is really interesting to improve freshness and complexity. The use of two non-*Saccharomyces* species enhances the floral notes and fruitiness and *S. cerevisiae* guarantees the suitable end of fermentation. Moreover, when an acidifier yeast is used, such as *L. thermotolerans*, the wine freshness is also enhanced.

## 6. Non-*Saccharomyces* and Off-Flavour Production

Undesired aroma compounds could be produced during fermentation by non-*Saccharomyces* yeasts (Table 3). These volatile compounds comprise large amounts of volatile acidity, sulphur compounds, excessive fusel alcohol aromas, etc. Among yeast strains known as sulphydric acid producers (H_2_S), as in the case of *T. delbrueckii* strains [82], some *M. pulcherrima* isolates have been studied due to their low ability to produce such volatile compound [83]. The production of H_2_S has been studied in 9 strains of *T. delbrueckii*, all them ranked 3–4 in a 0 to 5 scale showing stronger production of this defective compound compared with *S. cerevisiae* and other oenological non-*Saccharomyces* yeasts [57]. Other odd smells related to the metabolism of *M. pulcherrima* are produced by aliphatic carboxylic acids such as hexanoic acid and octanoic acid [84]. The aroma associated to these compounds resembles fatty and cheese for the first acid, and rancid or harsh for the second one.

Apiculate yeasts with high prevalence in grape microbiome are usually described as high producers of volatile acidity. When the production is measured in pure fermentation of 11 strains of different species, some strains of *H. osmophila*, *H. valbiensys* and *H. uvarum* generated 0.6-0.8 mg/L acetic acid. However, other strains of these species, and also *H. vineae*, produced 0.4-0.5 mg/L [85]. Therefore, specific selection can help to obtain strains with suitable levels of volatile acidity. Many *Hanseniaspora* species frequently show high values of ethyl acetate ranging from 50 to >300 mg/L [85]. 

## 7. Biological Control of Indigenous Yeasts Producing Defective Off-Flavours

Biological control by yeasts is a powerful bio-tool to control spoilage indigenous populations which produces off-flavours and metabolites affecting wine quality and freshness. Several antimicrobial activities have been described in yeasts such as the production of pulcherrimin, killer factors, etc. (Table 4). 

*Metschnikowia fructicola* is used and commercialised for biological control of apiculate yeasts (*K. apiculate*/*H. uvarum*) reducing the formation of volatile acidity at the early stages of fermentation. *M. fructicola* produces Killer factor K2 which increases membrane permeability, that reduces respiration activity and lowers intracellular ATP content decreasing cell viability [89]. Several volatile compounds produced by *Hanseniaspora uvarum* have been described as effective inhibitors against the development of *Botrytis cinerea* [90] and, among them, *trans*-cinnamaldehyde showed the stronger inhibition of mycelium growth also avoiding conidia germination [91]. 

The species *Wickerhamomyces anomalus* has both properties conferred, biocontrol agent and antimicrobial agent due to its activity against moulds in diverse environments and to the production of killer toxins vs. spoilage yeast [92]. In the last decade, a *Torulaspora delbrueckii* killer strain was successfully isolated and its fermentative performance was assessed against *S. cerevisiae* [45]. This killer strain prevailed over *S. cerevisiae*, but only under conditions of must sterility or with high inoculum population. This biocontrol tool is interesting to ensure the metabolic prevalence of non-*Saccharomyces* yeasts *versus* the native must yeasts.

## 8. Emerging Technologies to Improve the Implantation of Non-*Saccharomyces*

A suitable expression of metabolites during fermentation, and therefore a significant sensory effect, depends on the implantation of the non-*Saccharomyces* yeasts used. The influence of the wild initial population, usually around 4-log CFU/mL of yeasts and 2-log CFU/mL of bacteria, is a determining factor for the successful implantation of the desired selected yeast. Especially when the non-*Saccharomyces* to implant is a weak fermenter (*M. pulcherrima*, *H. vineae*, *L. thermotolerans,* etc.) or it has slow fermentative kinetics (e.g., *S. pombe*). The reduction or elimination of the wild microbiota in the grape is a way to promote the implantation of non-*Saccharomyces* species, and therefore, to better express their metabolome producing a significant impact in the sensory profile. 

The use of non-thermal technologies provides a way to eliminate or control the indigenous population. At the same time, the sensory profile of the grape is preserved and thermal degradation of pigments and aromatic compounds or undesired oxidations are avoided. Some of these technologies can be highly effective in the control of microorganisms, and at the same time extremely gentle with the sensory components [32]. Most of the technologies affect the structure of skin cells facilitating the extraction of tannins, anthocyanins and aroma compounds. The use of High Hydrostatic Pressure (HHP), Ultra High Pressure Homogenization (UHPH), Pulsed Electric Fields (PEF), Ultrasound (US), β-irradiation (βi), Pulsed Light (PL) and Ozonation, favour the control of indigenous yeasts in grape and musts promoting the implantation of weak yeast starters of non-*Saccharomyces*. 

HHP has demonstrated a high efficiency eliminating yeast in crushed grapes reaching 4-log inactivation [100]; however, lactic acid bacteria populations were partially reduced too, and only 1-log remained viable even at 550 MPa-10 min. Several non-*Saccharomyces*: *S. pombe*, *T. delbrueckii*, *M. pulcherrima* and *L. thermotolerans*, showed better implantation when grapes are processed at 400 MPa-10min [73]. In addition, HHP increases the extraction of phenols, especially anthocyanins, facilitating faster macerations and wines with higher colour intensity [100,101,102]. 

UHPH is also a highly efficient technology to eliminate indigenous microorganisms from musts and, conversely to HHP, it is also extremely efficient against bacteria. Initial populations of 6-log CFU/mL yeast and 4-log CFU/mL aerobic and lactic acid bacteria were undetected in 1 mL after continuous processing of the must at 300 MPa [103]. Additionally, UHPH can inactivate oxidative enzymes favouring the reduction of SO_2_ levels [30]. 

PEF produces cell wall poration causing microbial inactivation [104], and it also promotes the extraction of phenolic compounds from the cell wall of grape’s skins, especially anthocyanins. The pre-fermentative use of 10 kV/cm increased anthocyanin content and wine colour intensity, and 5 kV/cm affected total polyphenol extraction [105]. PEF technology can be used continuously at industrial scale to facilitate maceration [75,106,107]. The use of PEFs to control microbial populations requires higher intensities, frequently >30 kV/cm [108]. 

Ultrasound irradiation is also an interesting technique to increase the extraction of phenolic compounds from grapes. However, the antimicrobial efficiency is reduced and it is associated to the thermal effect which produces degradation of sensory quality. 

PL is another highly effective non-thermal technology with the ability to destroy vegetative and sporulate forms of microorganisms. The application can be done in a continuous way over the destemmed grape when is selected in a sorting table. The use of this technology can help reducing SO_2_ levels and improving the implantation of non-*Saccharomyces*, as well as performing yeast-bacteria co-inoculations [109].

## 9. Cell Wall Polysaccharides from Non-*Saccharomyces* Yeasts

The ageing on lees (AOL) process consists in a long contact of lees with wine during the ageing period. The lees are formed essentially by yeast biomass. This technique is traditionally used in the elaboration of sparkling wines after the second fermentation in the bottle [110]. During the AOL, the yeast autolysis is produced. This phenomenon involves the decomposition of the cell membranes and the release of intracellular compounds to the wine. In addition, the cell wall is degraded by the action of enzymes produced by the dead yeast [111]; this includes the mannoproteins, the major cell wall polysaccharide. A major drawback of this technique is the time needed for the autolysis to be completed and the mannoproteins to be released which, in most cases, is of several months under conventional oenological conditions [25,26,112]. Research studies using different techniques to speed up this process include ultrasounds [28,29] and pulsed electric fields [113,114,115]. In this way, the time required for the AOL is shorten.

The different compounds released during yeast autolysis have a direct organoleptic repercussion in the wine aged. The amino acids and peptides act as flavour precursors and malolactic fermentation promoters [116]. A lipid fraction is also released to the wine during AOL; these compounds seem to have an impact on the foaming properties [117]. Regarding the wall polysaccharides, the mannoproteins work against tartaric and protein precipitations [118]. Polysaccharides decrease the astringency perception [119] and increase the body of the wine aged. Finally, the mannoproteins provide a higher monomeric anthocyanins stabilization [120]. 

The AOL could also impact the freshness perception of wines in terms of acidity and volatile fraction. The AOL seems not to have a direct influence on the acidity of aged wines. A decrease of tartaric acid after AOL has been observed [121], nonetheless, no significant differences were detected in the rest of the acids and titratable acidity. Other authors showed only minor differences in total acidity after 180 days of AOL in Chardonnay white wines [122]. 

The repercussion of the AOL on the aromatic fraction has been also studied. It has been observed that the AOL significantly increases the volatile compounds in wines from Bombino bianco grapes [123], and it was also observed that after long AOL (30 months), the wines from different grape varieties do not change their varietal aromatic characteristics [124]. In this regard, other aromatic compounds related to freshness perception such as volatile thiols, terpenes, acetate esters of higher alcohols or ethyl esters of short chain fatty acids, have been under study. Volatile thiols (4-methyl-4-mercaptopentanone and 3-mercaptohexanol) were analysed in wines after eight months of AOL in oak barrels [125]. The concentration of these compounds was larger in AOL wines. Therefore, it existed a protective effect on typical fruity aromas found in young wines. This effect could be explained by the increase of the sulphur tripeptide glutathione that play a major role in protecting volatile thiols during the aging of bottled white wines [125]. Terpenoid compounds, important contributors to varietal aroma of wines because of their low perception threshold and their relation with floral odour, increased their content in wines after ten months aged on lees [126]; α-terpineol, *E*-nerolidol and *Z*-nerolidol, as an example, significantly increased in Airén variety after contact with lees during ageing [124]. This effect might be possible due to the release of β-glucosidases during the yeast autolysis. Esters, compounds responsible in large part for the fresh and fruity aroma of wine, experienced a significant increase after the AOL of Chardonnay wines [126]. This increase was observed in Airén wines as well [124]. Nonetheless, besides that increase in the concentration of esters during ageing, some of these compounds could be absorbed by the yeast lees after long periods [127]. 

The nature of the cell wall of the different yeast species is different, therefore the use of non-*Saccharomyces* in AOL technique will result in wines with different chemical and organoleptic composition. The use of *Torulaspora delbrueckii* and other yeasts as *Saccharomycodes ludwigii* and *Zygosaccharomyces bailii*, both considered spoilage yeasts, can increase the polysaccharides content in wines, especially when spoilage yeasts are used [128]. Other yeast species such as *Schizosaccharomyces pombe* and *Saccharomycodes ludwigii* can also modify the polysaccharides content in wine [25]; an increase of ten-folds, in comparison to *S. cerevisiae*, were observed in wines after only 28 days of ageing when using both species, *S. pombe* and *S. ludwigii*; despite these observation, better results were obtained with *S. pombe*, *S. ludwigii* and *D. bruxellensis* compared to the control *Saccharomyces* strain [26]. 

## 10. Conclusions

If the 20th century was the time of *S. cerevisiae*, then the 21st is the time of non-*Saccharomyces* yeasts. The applications and commercial relevance of these species is becoming increasingly important to develop new opportunities to improve wine’s quality. Today’s research lines are mainly focused on the use of non-*Saccharomyces* yeasts to enhance sensory quality, including wine aroma, colour and structure. To describe wine’s quality and stability, is to talk about molecules with sensory repercussion or microbiological and physicochemical properties that are highly influenced by pH variations. The natural production of organic acids by some non-*Saccharomyces* yeasts helps to get safer and more stable wines even during barrel and bottle ageing. Moreover, the biocontrol properties of these yeasts enables the safe production of wines by the reduction of the content of wine preservatives such as sulphites. In this regard, the use of emerging non-thermal physical technologies would also become the basis for the new oenology with low use of chemical additives and a more controlled fermentative microbiota. 

## Figures and Tables

**Table 1:** Main metabolites of non-*Saccharomyces* yeasts, sensory repercussion and technical impact.

Non-*Saccharomyces* Species	Metabolite/ Biopolymer	Structure	Sensory Repercussion	Technical Impact	Sensory Olfactive Threshold (µg/L)^1^ * If usually above This Value	Reference
*Hanseniaspora/Kloeckera*	2-Phenylethyl acetate	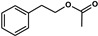	Floral, rose petals hints	Enhance floral notes x2-10 compared to *S. cerevisiae*	250 *	[35,37]
	Mannans		Cell wall polysaccharides, mannoproteins	Increased mouthfeel, even perceptible after fermentation		[50]
*Hanseniaspora vineae*	Benzyl acetate	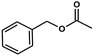	Floral jasmine aroma	Floral	2 *	[35,51]
*Lachancea thermotolerans*	2-Phenylethyl acetate	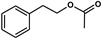	Floral, rose petals hints	10–50 mg/L	250 *	[15]
	Ethyl lactate	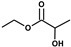	Strawberry, toffee	>40 mg/L High sensory threshold	150,000	[15]
	Lactic acid	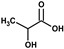	Citric acidity	0.3–16 g/L Up to 0.5 pH reductions in oenological conditions Slight sugar depletion with some alcohol reduction		[15,52]
*Metschnikowia pulcherrima*	2-Phenylethanol	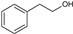	Rose-like odour	>30 mg/L	14,000 *	[48]
	Monoterpens (e.g., linalool)	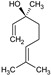	Floral	Increase varietal aromas by hydrolysing glucoside terpenes	25 *	[48]
*Pichia kluyveri*	Mercaptohexanol (3-MH)	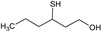	Grapefruit, passion fruit	Fruity smell: > 625 ng/L single fermentation to 3000 ng/L co-inoculation	0.060 *	[46]
	Mercaptohexyl acetate (3-MHA)	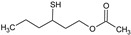	Grapefruit, passion fruit	Fruity smell: > 500 ng/L single fermentation to 1700 ng/L co-inoculation	0.004 *	[46]
*Schizosaccharomyces pombe*	Pyruvate	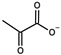	Stable pigments, colour stability Precursor for vitisin A type compounds: 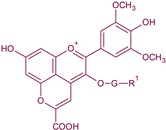	Enhance the formation of vitisin A derivatives Some strains also vinylphenolic pyranoanthocyanins		[53]
	Cell wall polysaccharides, mannoproteins		Better wine structure, softening of the astringency	Increased mouthfeel		
*Torulaspora delbrueckii*	2-Phenylethyl acetate	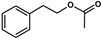	Flower, honey	1.2-2x compared to *S. cerevisiae* & *S. uvarum*	250 *	[42,44]
	Ethyl hexanoate	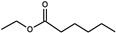	Apple	Fruity smell	62 *	[44]
	3-Ethoxy-1-propanol	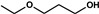	Black currant, solvent	Black fruity smell		[44]
*Wickerhamomyces anomalus*	2-phenylethyl acetate	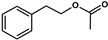	Flower, honey		250 *	[42]
	Isoamyl acetate	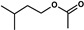	Banana	Enhance fruitiness	30 *	[42]
	Ethyl acetate	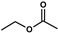	Fruity at low concentration	Fruity smell at low concentration Enhance complexity	12,300 *	[54]

^1^ Odour thresholds [42,54]; * It highlights all compounds which concentration is usually above the threshold value.

**Table 2:** Non-*Saccharomyces* species commercially available and main applications in wine production.

Non-*Saccharomyces* Species Commercially Available	Brand, Producer, Year, Format	Sensory Repercussion	Fermentative Performance (% vol. Ethanol) Recommended Inoculation Dose	Application. Requirements.	Reference
*Hanseniaspora vineae*	OENOBRANDS Launch 2021 Dry yeast	Enhance production of fruity and floral esters *De novo* formation of floral esters from sugars Increased body, softness and roundness	10%	Low SO_2_ Nutrition: thiamine and yeast extract	http://www.oenobrands.com/en/our-innovation
*Lachancea thermotolerans*	CONCERTO^TM^ CHr HANSEN 2012 Dry yeast	Red and black fruit integration Round mouthfeel Soft acidification Low volatile acidity, H_2_S and SO_2_	10% 25 g/HL	Red/White wines from warm areas	https://www.chr-hansen.com/
	LEVEL^2^ LAKTIA™ LALLEMAND 2018 Dry yeast	Enhancement of aromatic complexity, freshness and acidity Lactic acid production Low volatile acidity High glycerol production	<10% 25 g/HL	High nitrogen requirements Free SO_2_ must be <15 mg/L	https://www.lallemandwine.com/
*Metschnikowia fructicola*	Gaïa™ LALLEMAND 2016 Dry yeast	Improvement of the sensory expression Preservation of varietal character	Low to no fermentative power 7–25 g/HL	Reduce the use of SO_2_ and volatile acidity Active K2. Biological control Resistant to low pH and 50 mg/L SO_2_	https://www.lallemandwine.com/
*Metschnikowia pulcherrima*	LEVEL^2^ FLAVIA^®^ MP346 LALLEMAND 2013 Dry yeast	Release of varietal aromas	9% 25 g/HL	Specific enzymatic activity helping in releasing varietal aromas (terpenes and thiols) Free SO_2_ must be <15 mg/L	https://www.lallemandwine.com/
	LEVULIA^®^ PULCHERRIMA AEB Dry yeast	High production of 2-phenyl and isoamyl acetates and terpenes Low volatile acidity	11.5% 20–50 g/HL	Release of varietal aromas (terpenes)	https://www.aeb-group.com/
*Pichia kluyveri*	FROOTZEN^®^ CHr HANSEN 2010 Frozen yeast	Enhancement of volatile thiols Blackcurrant, liquorice, black pepper, menthol aromas	4–5% 1 bag/10kL	Enhance fruitiness	https://www.chr-hansen.com/
*Torulaspora delbrueckii*	PRELUDE^TM^ CHr HANSEN 2009 Frozen yeast	Enhance production of medium chain fatty acid esters Increased body, softness and roundness	9% 25 g/HL	Enhance fruitiness Promote malolactic fermentation by depletion of medium chain fatty acids	https://www.chr-hansen.com/
	LEVEL^2^ BIODIVA™ TD291 LALLEMAND 2009 Dry yeast	Aromatic complexity, ester production Low volatile acidity	10% 25 g/HL	Tolerance to osmotic pressure. Adapted for fermenting late harvest and ice wines Free SO_2_ must be <15 mg/L	https://www.lallemandwine.com/
	ZYMAFLORE^®^ Alpha LAFFORT 2010 Dry yeast	Aromatic complexity and good mouthfeel Suitable for making expressive and full-bodied wines Revelation of thiol-type varietal aromas (3MH, 3MHA)	10% 25 g/HL	Medium nitrogen requirements Low volatile acidity, volatile phenols and H_2_S	https://laffort.com/en/
*Torulaspora delbrueckii +* *Saccharomyces* spp.	Oenoferm^®^ wild & pure F3 Erbslöh Dry yeast	Enhanced mono terpenes and formation of fruity esters Support the ripe and exotic fruit aroma Full-bodied wines	- 20–40 g/HL	Moderate to high nitrogen requirement High alcohol tolerance	https://erbsloeh.com/en/
*Torulaspora delbrueckii +* *Metschnikowia pulcherrima*	ZYMAFLORE^®^ ÉGIDE LAFFORT 2017 Dry yeast	Organoleptic neutrality and control the microflora	10% 2–5 g/HL	Bioprotection of grapes and juices Restriction of the growth of indigenous flora SO_2_ reduction	https://laffort.com/en/
*Schizosaccharomyces pombe*	ProMalic^®^ PROENOL Dry beads	Wine deacidification	100g/HL	Maloalcoholic fermentation Free SO_2_ must be <14 mg/L	https://www.proenol.com/

**Table 3:** Non-*Saccharomyces* yeast species involved in the production of undesired volatile compounds in wines.

Non-*Saccharomyces* species	Metabolite	Structure	Off-Smell/Off-Flavour	Technical Impact. Sensory Threshold (µg/L) ^1^	Reference
*Hanseniaspora/Kloeckera*	Acetic acid	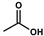	Vinegar taste	Many species/strains >0.6 g/L of volatile acidity 300,000	[85]
	Ethyl acetate	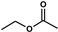	Solvent smell, nail varnish	Many species/strains >100 mg/L 12,300	[85]
*Lachancea thermotolerans*	Lactic acid	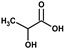	Some strains can produce excessive sour taste	Some strains >7 g/L; maximum described 16 g/L	[15,52]
*Metschnikowia pulcherrima*	Acetic acid	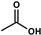	Vinegar taste	>0.7 g/L 300,000	[86]
	Ethyl acetate	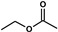	Solvent smell, nail varnish	>200 mg/L 12,300	[84]
	Hexanoic acid	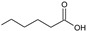	Fatty, cheese	>1.1 mg/L 420	[84]
	Octanoic acid	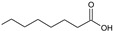	Rancid, harsh	>1.2 mg/L 500	[84]
*Schizosaccharomyces pombe*	Acetic acid	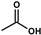	Vinegar taste	Many strains >1 g/L of volatile acidity 300,000	[62,86]
*Torulaspora delbrueckii*	Sulphydric acid	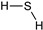	Reductive smell, egg rot smell	Impact depending on the intensity 1.1	[57,82]
*Wickerhamomyces anomalus*	Ethyl acetate	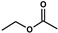	Solvent smell, nail varnish	≈100 mg/L 12,300	[85,87]
	Acetic acid	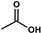	Vinegar taste	0.02 g/L 300,000	[86,87,88]

^1^ Odour thresholds [42,54].

**Table 4:** Non-*Saccharomyces* yeast species involved in the production of biocontrol agents.

Non-*Saccharomyces* Species	Metabolite	Antimicrobial Effect	Technical Impact	Reference
*Hanseniaspora uvarum*	*trans*-Cinnamaldehyde	Inhibition of mycelium growth and conidia germination	Biocontrol of *Botrytis cinerea*	[90,91]
*Metschnikowia pulcherrima*	Pulcherrimin	Iron depletion	Biological control Effective inhibitory activity against several yeasts: *Candida tropicalis*, *Candida albicans*, *Brettanomyces*/*Dekkera, Hanseniaspora* and *Pichia* genera; and some fungi: *Botrytis cinerea*, *Penicillium* spp., *Alternaria* spp. and *Monilia* spp.	[8,93,94,95,96,97,98]
	Killer factor	Membrane permeabilization	Biological control	[57]
*Metschnikowia fructicola*	Killer factor: active K2	Increase of membrane permeability	Biological control of apiculate yeasts: *K*. *apiculate*/*H. uvarum* Reduction of volatile acidity	www.lallemandwine.com/
*Wickerhamomyces anomalus*	Exo-β-1,3 glucanase	Wall-lytic enzymes	Inhibition of *Botrytis cinerea*	[92]
	Pikt killer toxin	Mycocins that control apiculate wine yeasts	Biological control of *Dekkera/Brettanomyces* spp.	[92,99]
*Torulaspora delbrueckii*	Kbarr-1 killer toxin	Toxicity against sensitive strains of *S. cerevisiae*	Better implantation and domination of the fermentation on the native *S. cerevisiae* of the must	[45]
